# Prospective study of toric IOL outcomes based on the Lenstar LS 900® dual zone automated keratometer

**DOI:** 10.1186/1471-2415-12-21

**Published:** 2012-07-16

**Authors:** Kjell Gunnar Gundersen, Richard Potvin

**Affiliations:** 1 Privatsykehuset Haugesund, Haraldsgaten 139, Haugesund, 5527, Norway; 2 Science in Vision, 13860 E. Riviera Dr., Burleson, Texas, 76028, USA

**Keywords:** Toric IOL, Astigmatism, Cataract surgery, Cataract surgery planning, Keratometer, Lenstar LS 900®

## Abstract

**Background:**

To establish clinical expectations when using the Lenstar LS 900® dual-zone automated keratometer for surgery planning of toric intraocular lenses.

**Methods:**

Fifty eyes were measured with the Lenstar LS 900® dual-zone automated keratometer . Surgical planning was performed with the data from this device and the known surgically induced astigmatism of the surgeon. Post-operative refractions and visual acuity were measured at 1 month and 3 months.

**Results:**

Clinical outcomes from 43 uncomplicated surgeries showed an average post-operative refractive astigmatism of 0.44D ±0.25D. Over 70% of eyes had 0.50D or less of refractive astigmatism and no eye had more than 1.0D of refractive astigmatism. Uncorrected visual acuity was 20/32 or better in all eyes at 3 months, with 70% of eyes 20/20 or better. A significantly higher number of eyes had 0.75D or more of post-operative refractive astigmatism when the standard deviation of the pre-operative calculated corneal astigmatism angle, reported by the keratometer, was > 5 degrees.

**Conclusions:**

In this single-site study investigating the use of the keratometry from the Lenstar LS 900® for toric IOL surgical planning, clinical outcomes appear equivalent to those reported in the literature for manual keratometry and somewhat better than has been reported for some previous automated instruments. A high standard deviation in the pre-operative calculated astigmatism angle, as reported by the keratometer, appears to increase the likelihood of higher post-operative refractive astigmatism.

## Background

Modern small-incision phacoemulsification cataract surgery provides patients with a quick and effective resolution of their cataract symptoms. As technology has improved, so has the ability to predict clinical outcomes [1]. There is now a high likelihood of a refractive result near plano for a majority of patients [2]. For those patients with significant corneal astigmatism, recent studies have demonstrated that implantation of a toric intraocular lens increased the likelihood of lower post-operative refractive astigmatism relative to a spherical monofocal IOL [3,4]. The percentage of patients with corneal astigmatism who did not require spectacles for distance vision was also high.

There are a number of factors that contribute to a successful clinical outcome with a toric intraocular lens. During the planning process the accurate measurement of ocular biometry is critical, particularly with regard to corneal astigmatism [5]. Appropriate adjustment for expected surgically induced astigmatism will also affect results [6]. The ability to properly align the lens at the time of surgery is important, as is rotational stability after implantation [7,8].

The study described here is related to the measurement of corneal astigmatism. When the AcrySof® Toric (Alcon, Fort Worth, TX) intraocular lens was introduced, the recommendation was that manual keratometry be used to measure corneal astigmatism. A recent retrospective analysis of results from a variety of keratometry devices suggested that clinical outcomes equivalent to manual keratometry could be achieved with these alternative devices [5]. A similar retrospective study was conducted between manual keratometry and dual-zone automated keratometry (Lenstar LS 900®, Haag-Streit AG, Koeniz, Switzerland). This latter study included simulation of results based on the different pre-operative measurements of corneal astigmatism from different devices [9]. By design, the simulation included the surgical variability expected on a case by case basis.

The current study was designed as a follow-up to the last study described above. It was a prospective single-arm study to determine the distribution of post-operative refractive errors with a toric intraocular lens when keratometry data from the Lenstar LS 900® was used for the pre-operative measurement on which surgical planning was based. Of additional interest was whether there was a variability factor that might be used to identify eyes where a less predictable result might be expected. For this latter purpose the standard deviations of the keratometric astigmatism magnitude and axis, as measured and reported by the Lenstar LS 900®, were the variables examined.

## Methods

This single-arm study provided one intervention: use of the dual-zone autokeratometry feature of the Lenstar LS 900® biometer as the basis for toric IOL selection. This met the current standard of care in the study clinic so there was no additional risk to patients. Institutional Review Board approval was applied for and obtained before patients were enrolled (Southwest Independent Institutional Review Board, Inc., Fort Worth, TX). Patients signed an informed consent form permitting use of their de-identified data.

The Lenstar LS 900® uses 32 measuring points arranged in 2 concentric rings (outer 2.3 mm, inner 1.65 mm) of 16 measuring points each. Each displayed keratometry measurement is a composite of the mean of 4 measurements, totaling 128 measuring points. With the recommended 5 scans, the keratometry is therefore calculated on the basis of 640 measuring points. Once the data are captured, the spherical equivalent radius is calculated for each individual measuring point. The keratometric calculation considers the best-fit ellipsoid built by the reflected points to determine the radii of the circumscribed ellipsoid. Results are then expressed in dioptric or millimeter notation (Lenstar LS 900® Biometer, Instruction Manual, Section 4.3.2. Haag-Streit AG, Koeniz, Switzerland). In contrast, manual keratometry measurement generally consists of a single reading of the alignment of 2 perpendicular mires at a diameter of approximately 3.2 mm.

During the measurement process the software checks every image for the quality of the marker point representation and the reproducibility of the points. Plain text messages advise the user how to improve the measurement quality e.g. if eye lashes or low eye lids distort or even block markers, then the user is indicated to encourage the patient to open up the eyes wide. Additionally to these individual checks, all repetitive measurements are tested for their plausibility as compared to the other measurements on the same as well as on the adjacent eye. Anomalous measurements are flagged for review. As a final quality control the standard deviation of the repetitive measurements is displayed to the user. The standard deviation of the calculated angle of astigmatism is one measure of interest in this study.

A total recruitment of 50 eyes was planned for the study, with an AcrySof® Toric IOL (Alcon, Fort Worth, TX), implanted in each eye that was enrolled. These lenses correct from 1.5D to 6.0D of astigmatism at the IOL plane, corresponding to 1.03D to 4.11D at the corneal plane for a nominal eye. Surgical planning for the astigmatism power of the lens was completed using the online toric calculator provided for that purpose (http://www.acrysoftoriccalculator.com). The previously calculated surgically induced astigmatism (SIA) for the surgeon was used along with the keratometry readings from the Lenstar LS 900®. The SIA calculation was based on previous surgeries with the same incision size and orientation, to reduce potential variability related to these variables. Operative data included the IOL implanted and the axis at which it was implanted, along with the surgeon’s SIA and the incision angle for each surgery.

All study eyes were operated on by the same surgeon (KGG).

Just prior to surgery, the axis of astigmatism (as determined from the toric calculator) was marked directly by the surgeon. The first 21 eyes were manually marked with waterproof ink at the slitlamp, whereas the last 29 eyes were marked with waterproof ink using a handheld pendulum marking instrument (G-33776 by Geuder AG, Heidelberg, Germany).

Standard phaco surgery was performed in all study eyes. The main incision (2.2 mm width) was placed at the 12 o’clock position, with two side port incisions (1 mm width) placed at the 10 and 2 o’clock positions. All incisions were clear corneal at a 45° to 60° angle. A corneal ring of 5 mm was used as a guide to obtain a standardized capsulorhexis size and location centered around the visual axis. A 5 mm capsulorhexis size was chosen to obtain a 360° contact between the anterior capsule and the anterior surface of the IOL, to ensure maximal stability of the IOL inside the capsular bag.

After implantation of the AcrySof IQ Toric IOL in the capsular bag, the lens was rotated and aligned to the ink marks and finally pushed back to the posterior capsule to ensure proper and durable alignment and “fixation”. After instillation of intracameral antibiotics, stromal hydration of all incisions ended the procedure.

The primary end point was the post-operative refractive data at 3 months. Secondary end points were the post-operative keratometry and visual acuity. Data collection included pre-operative keratometry, along with keratometry, visual acuity and refractive data at 1 month and 3 months post-operatively.

Clinical data were tabulated and de-identified on case report forms, along with printouts from the AcrySof Toric Calculator and the Lenstar LS 900® biometer. Preliminary data checking and analysis was performed using Access database software (Microsoft Corp.). The preliminary analysis included calculating corneal astigmatism from keratometry values. Statistical analyses were performed using the Statistica data-analysis software system (version 9.1, Statsoft, Inc.).

The primary measure of interest was the distribution of residual astigmatism (magnitude and axis) for the patient population. Levels of uncorrected and best-corrected visual acuity were also of interest. A secondary measure of interest was whether the standard deviations of the keratometric astigmatism or keratometric axis were correlated to higher variability in refractive outcomes. For this last measure, statistical testing with appropriate parametric and non-parametric tests was conducted using a significant p value of<0.05.

## Results

A total of 50 eyes of 31 subjects were enrolled in the study; 19 subjects had both eyes enrolled. Calculated by eye, average age was 67.0 ±8.6 years (range 50 to 83). There were 25 female eyes and 25 male eyes. Average pre-operative corneal astigmatism was 1.90 ±0.64D (range 1.12 to 3.69).

Three eyes over the course of the study experienced complications that were likely to affect visual outcomes unrelated to surgical planning. One had persistent cystoid macular edema, one required a trabeculectomy due to a pressure spike between the 1 and 3 month visits and one eye experienced an episode of iridocyclitis. The data for these eyes were eliminated from the analysis. In four eyes the recommendation for the cylinder power of the toric IOL, calculated from the Lenstar LS 900® data, was modified. These eyes were also excluded from the analysis; the remaining 43 eyes were analyzed as a group.

Table 1 lists the IOLs implanted in the study, along with the average pre-operative keratometric astigmatism and the average post-operative refractive astigmatism for each IOL. While there are insufficient data by lens to draw a statistically significant conclusion, it can be seen that the average residual refractive astigmatism does not appear related to the magnitude of pre-operative corneal astigmatism. Grouping the data as SN6AT3, SN6AT4 and “Higher”, an ANOVA of the residual refractive astigmatism by lens group showed no statistically significant difference (p > 0.8). Overall mean refractive astigmatism was −0.44D ±0.25D at both 1 and 3 months post-operative, and appeared stable. The vector difference in refractive astigmatism between 1 and 3 months was 0.25 +/− 0.2D, on the order of test-retest reliability for refractive data.

**Table 1:** Pre-operative corneal and post-operative refractive astigmatism by Toric Lens Model

			**Postoperative Refractive Astigmatism**					
Lens Model*	**Count**	**Pre operative Avg Corneal Astigmatism**	**1 Month**			**3 Months**		
			**Avg**	**Min**	**SD**	**Avg**	**Min**	**SD**
SN6AT3 (1.03)	20	1.45	−0.49	−1.00	0.30	−0.46	−1.00	0.26
SN6AT4 (1.55)	12	1.84	−0.38	−0.75	0.23	−0.42	−0.75	0.27
SN6AT5 (2.06)	62	2.48	−0.29	−0.75	0.29	−0.42	−0.75	0.30
SN6AT6 (2.57)	3	2.85	−0.50	−0.75	0.25	−0.42	−0.75	0.29
SN6AT7 (3.08)	2	3.61	−0.63	−0.75	0.18	−0.50	−0.50	0.00

*Figure in brackets is the normal cylinder correction (D) at the corneal plane for an average eye.

The standard deviation of the angle of astigmatism was also compared by lens type (it is not unusual for the axis to be more difficult to reliably determine when astigmatism is low); there was no statistically significant difference in the standard deviation of the angle of astigmatism (p > 0.2).

**Figure 1:**
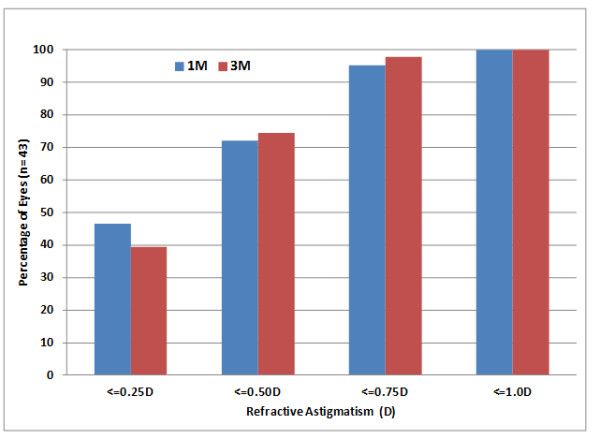
Cumulative distribution of post-operative refractive astigmatism.

Figure 1 shows a histogram of the post-operative refractive astigmatism for the 43 eyes analyzed as a group. As can be seen over 70% of eyes had 0.50D or less, and no eye had more than 1.0D, of post-operative refractive astigmatism.

The pre-operative and post-operative keratometry data from the Lenstar LS 900® provided a means to calculate the actual surgically induced astigmatism (SIA) by making a vector subtraction of the post-operative corneal astigmatism at 3 months from the pre-operative corneal astigmatism. There were 45 eyes with appropriate keratometry data for the calculation. The SIA value used for toric IOL calculations in the planning process was 0.3D. The calculated mean SIA value was 0.47D ±0.42D (range 0.08 to 2.75D). Only one eye had a calculated SIA > 1.2D (2.75D). This eye had a chalazion removed 3 weeks post-operatively but it is unclear whether that had an effect. This was also the eye that had the greatest amount of residual refractive astigmatism. When this one significant outlier was excluded from the SIA analysis the mean calculated SIA was 0.42D ±0.25D (range 0.08 to 1.12). There was no evident preoperative factor that would have distinguished this eye from others in the study.

**Figure 2:**
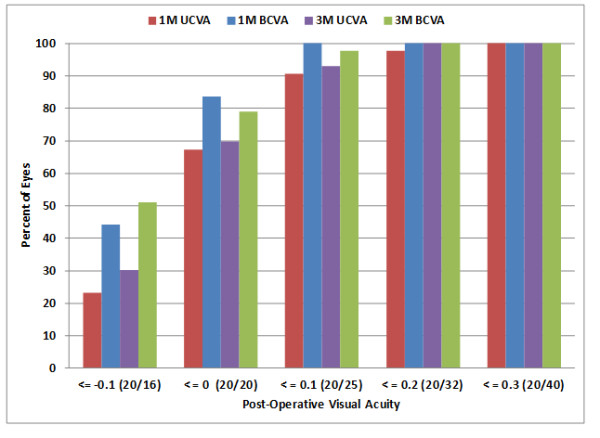
Cumulative distribution of post-operative visual acuity.

Figure 2 shows the cumulative uncorrected and best-corrected visual acuity after cataract surgery at 1 and 3 months post-operatively. Almost 70% of eyes had uncorrected visual acuity of 20/20 (logMAR 0.0) or better; fewer than 85% of eyes could be corrected to this level. All eyes but one were 20/32 (logMAR 0.2) or better at 1 month and all eyes were 20/32 or better at 3 months in both the uncorrected and corrected states.

There was the potential that surgical technique or the surgeon’s toric alignment would improve over time. The change in marking methods might also have had an effect. A test of residual refraction by date of surgery indicated no systematic difference in clinical results (p > 0.05).

The standard deviation (SD) from each of the two K readings and the astigmatism angle were available from the Lenstar LS 900® printout. A correlation matrix that included the post-operative refractive astigmatism, the K readings, the magnitude and angle of the corneal astigmatism and the given standard deviations was created. There was no statistically significant correlation observed between any of these variables.

Taking a more categorical approach, the SD of the angle of astigmatism calculated was used to divide the eyes, with a SD of 5 degrees or more being considered high and less than that to be normal. Of the 4 eyes in the high group, 3 (75%) had refractive astigmatism >0.50D at 3 months, while in the normal group only 8 of the 39 eyes (20%) had refractive astigmatism >0.50D at 3 months. While there are few eyes in the high group, a Fisher’s exact test indicates that the difference is statistically significant (p <0.05).

Using the same approach, the K readings were categorized on the basis of the SD of the individual steep and flat K measurements. There were 10 eyes with a K SD of 0.2D or more (one or the other, only one eye had a K SD > 0.2 for both steep and flat values). Only 2 of these 10 eyes had refractive astigmatism >0.50D at 3 months, while 9 of the remaining 33 eyes had that level of astigmatism. The difference was not statistically significant (Fisher’s exact test, p >0.5).

## Discussion

Overall results for this series of patients where keratometry from the Lenstar LS 900® was used for surgical planning of astigmatism correction were equivalent or better than that achieved in the clinical study conducted for FDA approval of the lens [AcrySof® IQ Toric IOL Package Insert], where manual keratometry was used for astigmatism planning. More than 70% of eyes in the current study had 0.50D or less of refractive astigmatism post-operatively, compared to the 63% reported in the package insert. More than 65% of eyes had 20/20 (logMAR 0.0) uncorrected acuity post-operatively, compared to 40% reported in the package insert. The populations were slightly different, as the magnitude of astigmatism correction that can be achieved has increased with the expansion of the IOL line. However, with all but 5 patients in the same range as the original study, and no observed effect of pre-operative corneal astigmatism on post-operative outcomes, this is unlikely to have been a major factor.

These results corroborate the earlier simulated analyses for the keratometry data from the Lenstar LS 900® [9]. The conclusion from that study was that clinical results from surgical planning based on the Lenstar LS 900® were likely to be equivalent to those achieved using manual keratometry.

Results with the Lenstar LS 900® are also consistent with previous toric IOL studies in the literature, some of which also used automated devices for keratometry readings. Ahmed et al. reported 71% of 164 eyes with 0.50D or less of refractive astigmatism at 6 months post-operatively, very similar to results reported here [4]. Automated keratometry was most often used in that study, though manual keratometry was used at some sites.

Results were significantly better than reported for toric IOLs implanted on the basis of data from a different automated biometer [10]. In that previous study, uncorrected visual acuity in 30 eyes was reported as 20/25 (0.1 logMAR) or better in 67% of eyes, compared to the 90% reported here. Average refractive cylinder was 0.72D ±0.43D, significantly higher than the amount reported here (0.44D ±0.25D at 3 months post-operative).

There is a suggestion in the current data that a standard deviation in the astigmatism angle measurement of more than 5 degrees may be associated with higher levels of post-operative refractive astigmatism. There was no such correlation between the standard deviations of the individual keratometry readings (flat, steep) and post-operative astigmatism. This is not entirely unexpected. Determination of the curvature on a given meridian is a much simpler exercise than determining the best-fit ellipsoid over the corneal surface. The SD of the keratometry readings is related to variability in the former, the SD of the astigmatism angle is related to variability in the latter.

With regard to the association of higher levels of post-operative refractive astigmatism and high variability in the angle of astigmatism, the root cause cannot be established; correlation and causality are very distinct entities. Whether it is related to a series of suboptimal measurements on a relatively normal eye, or whether it is related to difficulties measuring an eye because of some abnormality, cannot be determined from the current data. This would merit additional research. However, even in the absence of this clarification the following recommendation can be made. If a high standard deviation is noted in the astigmatism angle, it is recommended that a second measurement be made to see if that variability can be reduced.

## Conclusions

This single-site study investigated the use of keratometry data from the Lenstar LS 900® dual-zone automated keratometer for toric IOL surgical planning. Clinical outcomes appear equivalent to those reported in the literature for manual keratometry and somewhat better than has been reported for some previous automated instruments. A high standard deviation in the calculated astigmatism angle (reported by the device) appears to increase the likelihood of higher post-operative refractive astigmatism.

## Competing interests

KGG has no competing interests.

RP is a consultant to Haag-Streit.

Haag-Streit AG provided funding for this research. Haag-Streit was not involved in the study design or analysis of results. The decision to submit the manuscript for publication was made by Drs. Gundersen and Potvin independent of any Haag-Streit input.

## Authors’ contributions

RP designed the study. KG provided the required clinical data. KG and RP reviewed the design, reviewed the results and wrote/reviewed the final paper. All authors read and approved the final manuscript.

## Pre-publication history

The pre-publication history for this paper can be accessed here:

http://www.biomedcentral.com/1471-2415/12/21/prepub
